# Multifrequency multi-qubit entanglement based on plasmonic hot spots

**DOI:** 10.1038/srep13941

**Published:** 2015-09-09

**Authors:** Jun Ren, Tong Wu, Xiangdong Zhang

**Affiliations:** 1 School of Physics and Beijing Key Laboratory of Nanophotonics & Ultrafine Optoelectronic Systems, Beijing Institute of Technology, 100081, Beijing, China

## Abstract

The theoretical method to study strong coupling between an ensemble of quantum emitters (QEs) and surface plasmons excited by the nanoparticle cluster has been presented by using a rigorous first-principles electromagnetic Green’s tensor technique. We have demonstrated that multi-qubit entanglements for two-level QEs can be produced at different coupling resonance frequencies, when they locate in the hot spots of the metallic nanoparticle cluster. The duration of quantum beats for such an entanglement can reach two orders longer than that for the entanglement in a photonic cavity. The phenomenon originates from collective coupling resonance excitation of the cluster. At the frequency of single scattering resonance, the entanglement cannot be produced although the single QE spontaneous decay rate is very big.

Quantum entanglement plays a key role in quantum information processing such as quantum teleportation^1^, quantum cryptographic^2^, quantum dense coding^3^ and parallel computing^4^. Entanglement between two particles has been well understood and used in different physical systems for a variety of tasks^5^,^6^,^7^,^8^,^9^. In contrast to the bipartite cases, the deep understanding on the multipartite entanglement is still in its infancy^9^. The generation of genuine multipartite entanglement has been demonstrated in a few physical systems such as ion traps^10^,^11^,^12^,^13^, photon systems^14^,^15^,^16^. Effective creation of large genuine multipartite entanglement with long duration of quantum beats is still a challenge.

On the other hand, surface plasmon polaritons (SPPs) have been subject of recent studies. Resonant excitation of SPPs allows metallic nanostructures to concentrate electromagnetic (EM) field in subwavelength volumes, resulting in enormous field enhancement^17^,^18^,^19^. For this reason, the interaction between quantum emitters (QEs) and SPPs has attracted great interest^20^,^21^. Up to now, many interesting theoretical and experimental works on such an interaction have been widely carried out^22^,^23^,^24^,^25^,^26^,^27^,^28^,^29^,^30^. For example, subradiance and superradiance resulting from the plasmon-mediated interaction have been demonstrated^20^,^31^, energy transfer between two QEs mediated by SPPs has been analyzed^32^,^33^. Recently, quantum entanglement generation between two separated QDs mediated by a plasmonic waveguide has been reported^34^,^35^,^36^,^37^,^38^,^39^. However, these investigations only focus on the bipartite entanglement.

Motivated by these recent developments in plasmonics and quantum information science, in this work we study the generation of multipartite entanglement based on the interaction between QEs and SPPs. Recent investigations have shown that the strong coupling between QEs and SPPs can appear when QEs are put in the nanogaps between adjacent metallic nanoparticles (so-called hot spots)^40^,^41^. Thus, our studies focus on the interaction between the nanoparticle cluster and QEs in the hot spots.

## Results and Discussion

We consider N two-level QEs located in the nanoparticle cluster. Let us further assume that the QEs are sufficiently far from each other, so that interatomic Coulomb interactions can be ignored. Under the electric-dipole and rotating wave approximations, the Hamiltonian of the system can be expressed as^24^,^31^,^42^
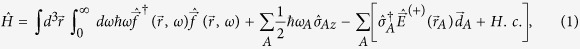
where 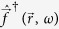 and 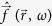 are referred to as the creation and annihilation operators of the radiation field, respectively. The 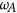 and 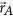 are the transition frequency and position vector of the Ath QE, 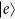 and 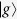 respectively represent its excited and ground states, 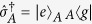, 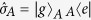, and 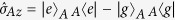 are Pauli operators, 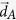 is the dipole moment. The electric field operator in Eq. (1) is^24^,^31^,^42^
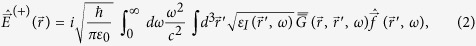
where 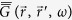 is the classical Green tensor of the system, which can be obtained exactly from the T-matrix method. Details of the calculated method are provided in the Methods section. Here 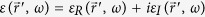 represents the complex permittivity. It is shown clearly from Eq. (1) that the interactions between the QEs and the SPPs have been realized without the laser pulse being introduced in the present systems, the QEs couple directly to the SPPs by themselves. For a single-quantum excitation, the system wave function at time t can be written as^31^,^42^
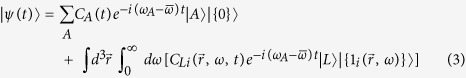
where 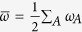, 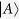 represents the upper state of the Ath QE and all the other QEs are in the lower state, 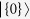 is the vacuum state of the medium-assisted field, 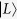 is the lower state of QEs and here 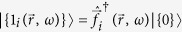 is the state of the field which is excited in a single-quantum Fock state, 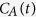 and 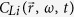 are the probability amplitudes of the states 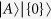 and 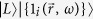.

According to the method described in ref. 31, if the new states 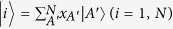 are introduced, at the same time the interference term of decay rate of any two QEs 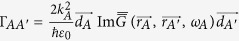 and the spontaneous decay rate of single QE 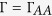 can be obtained, the Schrodinger equation based on Eqs (1 and 3) for the present system can be solved, namely the probability amplitudes 
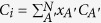
 can be obtained. Then, if one of these new states 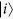 is in the strong coupling and other states are in the weak coupling, the corresponding spontaneous decay rate to 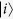, 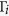, is very large and the decay rates for other states are nearly equal to zero. Then, the state 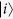 is often called superradiant state and other states are subradiant states. For these subradiant states, the probabilities vanish under the initial conditions 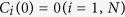. Thus, after tracing out the medium-assisted field, the density operator of N qubits can be written as
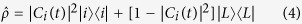


In fact, there are several methods to scale the multipartite entanglement, for instance, to analyze the pairwise entanglement^43^, calculate the entropy of entanglement between one part and the rest of the system^44^, and consider the global entanglement^45^. These methods all have their limitations. The genuine entanglement we used in the following is a revised form of the global entanglement, it is similar to the global entanglement but more general. According to ref. 46, the genuine multipartite entanglement is defined as
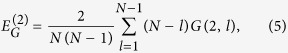
where the function *G*(2, *l*) is defined as
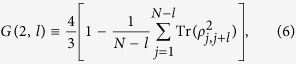
and 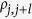 is the reduced density matrix of qubits *j* and *j*+*l*, which is obtained by tracing out the other *N*−2 qubits. Based on Eqs (4, 5, 6), we can calculate the genuine multi-qubit entanglement once 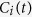 has been obtained.

### Two-qubit entanglement

**Figure 1:**
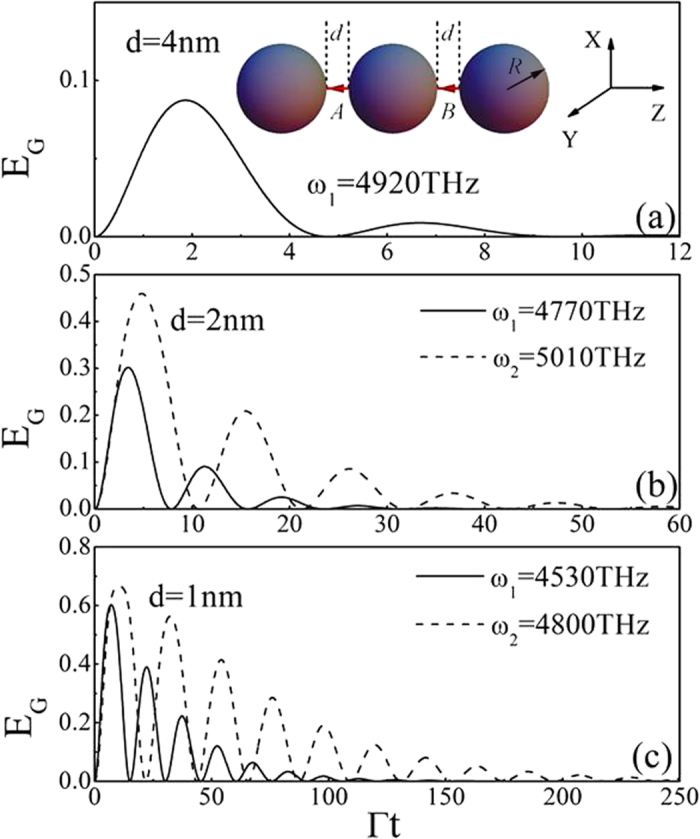
Genuine entanglement of two QEs inserted in the gaps of a linearly arranged silver nanosphere trimer (shown in inset) with various separation distances and frequencies: (**a**) d = 4 nm and *ω*_1_ = 4920 THz; (**b**) d = 2 nm, *ω*_1_ = 4770 THz(solid), *ω*_2_ = 5010 THz (dashed); (**c**) d = 1 nm, *ω*_1_ = 4530 THz (solid), *ω*_2_ = 4800 THz (dashed). The radii of nanospheres are R = 10 nm, and the orientations of electric dipole moments for two QEs are both along the axis of the trimer.

We consider two two-level QEs located at the hotspots of linear nanosphere trimer as shown in the inset of Fig. 1(a), the radii of three spheres are taken as R and the separation distances (gaps) between them are marked by d, two QEs A and B are inserted in the gaps, and the orientations of their electric-dipole moments 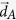 and 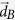 are both along the axis of the trimer. The new states 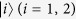 are taken as 
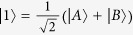
 and 
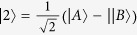
, their corresponding probability amplitudes are 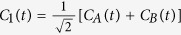 and 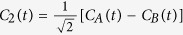, and the decay rates are 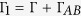 and 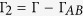. The excitation can initially resides in one of the QEs or the medium-assisted field. If the excitation resides in the medium-assisted field initially, that is 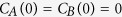 and 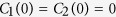, which can be realized by coupling the field first to another excited QE E with the probability amplitude 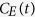 in a time interval Δ*t*. If the QE E locates at the same position with the QE A, and taking into account that A, B and E obey the same law of decay, from the Schrodinger equation, the corresponding integro-differential equation of 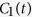 is^42^
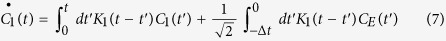
with 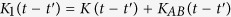, and 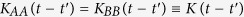, where
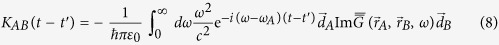


In the strong coupling regime, 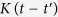 can be approximated as^47^
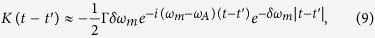
where 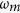 and 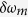 are the resonance frequency and linewidth of the system, respectively. Similarly,
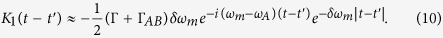


At the resonance frequency, 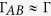 can be realized, there is 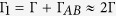, and suppose 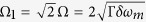, after differentiating Eq. (7) we arrive at
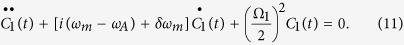


As 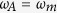, 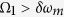 and the initial condition 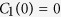, the solution of Eq. (11) is
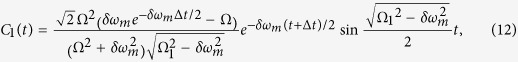


Following the same procedure with 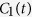, we can obtain
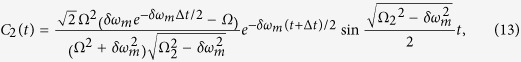
where 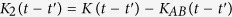, 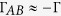 and 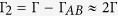 are taken. Here 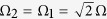 and the line-width 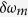 is determined by the imaginary part of the eigen-frequency^48^, which can be obtained by solving the eigenvalue of the system (see Methods section for the detailed process). Having obtained 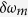 and Γ, the Rabi frequency Ω can also be calculated. In general, when 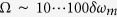, the strong coupling can be realized^42^. Our calculated results show that the strong coupling condition can be reached in above systems when the separation distance is small, like d = 1 nm. Then, from Eqs. (12 and 13) combining with Eqs (4, 5, 6), we can calculate the genuine entanglement for two-qubit system with 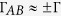.

Figure 1 shows the calculated results when two-level QEs located at the hot spots of a linearly arranged silver nanosphere trimer with various separation distances. The radii of silver spheres are taken as R = 10 nm. For the dielectric functions of Ag, the Johnson’s data were adopted (the absorption loss is included)^49^. Thus, the effect of decoherence has been considered. The parameters of the molecular dipole of the QEs are taken according to ref. 50: 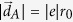 and 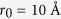. Figure 1(a–c) correspond to the genuine entanglement with d = 4 nm, d = 2 nm and d = 1 nm at different frequencies, respectively. Comparing them, we find that both the amplitude and duration of quantum beats for the genuine entanglement increase with the decrease of the separation distance d. For example, as d = 4 nm, the maximum value of 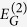 is less than 0.1, it reaches 0.63 at d = 1 nm. In Fig. 3 of ref. 35, the concurrence of two-qubit system in a photonic cavity decreases to less than 0.2 in a very short time interval 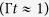, and in the present system, this duration of quantum beats can reach 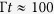
(dashed line in Fig.1(c)). That is to say, the duration of quantum beats for such a case can reach two orders longer than that for the entanglement in a photonic cavity (CQED). These results are for the genuine entanglement. In fact, we have used another popular method given in refs 51 and 52 to scale entanglement by calculating the concurrence of the present two-qubit system. Comparing the calculated results from two kinds of method, we find that they are identical.

Another feature is that the entanglement for two-level QEs can appear at different frequencies simultaneously with the decrease of d. As d = 4 nm, the entanglement only appears at one frequency *ω*_1_ = 4920 THz (Fig. 1(a)), it appears at *ω*_1_ = 4770 THz (solid line in Fig. 1(b)) and *ω*_2_ = 5010 THz (dashed line in Fig. 1(b)) simultaneously for the case with d = 2 nm, at *ω*_1_ = 4530 THz(solid line in Fig. 1(c)) and *ω*_1_ = 4800 THz (dashed line in Fig. 1(c)) for the case with d = 1 nm.

**Figure 2:**
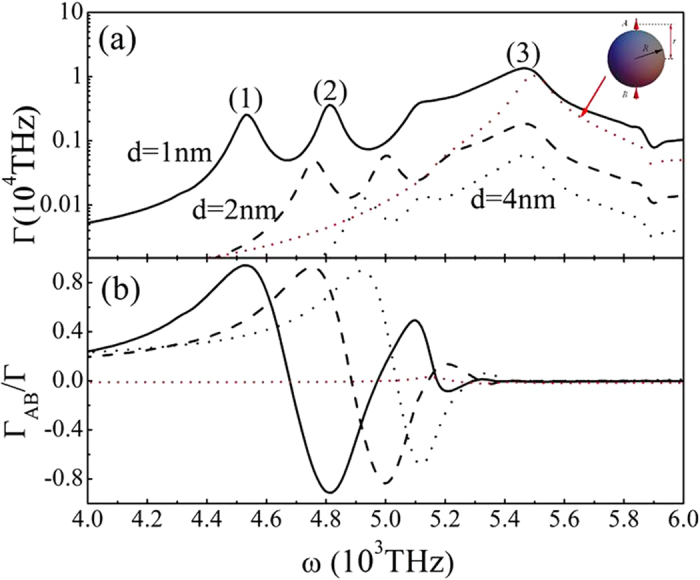
(**a**) Spontaneous decay rate Γ and (**b**) interference term 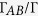 of QEs in the structures described in Fig. 1. The solid line, dashed line and dotted line correspond to the cases with d = 1 nm, 2 nm and 4 nm, respectively. The other parameters are identical with those in Fig. 1. Red dotted line represents the corresponding result for a single sphere and two QEs without hotspots as shown in inset.

In order to disclose the physical origin of the above phenomena, in Fig. 2(a,b) we plot the corresponding single QE spontaneous decay rate Γ and the interference term 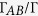 between QEs as a function of frequency at various gaps. The solid, dashed and dotted line correspond to the case with d = 1 nm, 2 nm and 4 nm, respectively. The value of decay rate Γ has a whole decrease with the increase of d. It is shown clearly that there exist three enhanced peaks (marked by (1), (2) and (3) for d = 1 nm) for each case. These peaks originate from two kinds of plasmon resonance, single scattering resonance and coupling resonance. The peak (3) comes from the single scattering localized surface plasmon resonance, which is determined by the property of the single sphere and is not sensitive to the gaps. Thus, it corresponds to the calculated result without hotspots (red dotted line), that is, two QEs locate at the two poles of the sphere as shown in the inset of Fig. 2(a). The other two peaks (peak (1) and (2)) are caused by the coupling plasmon resonances, which are very sensitive to the separation distances between two spheres. When the separation distances become very large, the coupling resonance peaks disappear and the phenomenon degenerates to the case of single sphere. With the decrease of the gaps, the peaks shift to longer wavelength (redshift).

**Figure 3:**
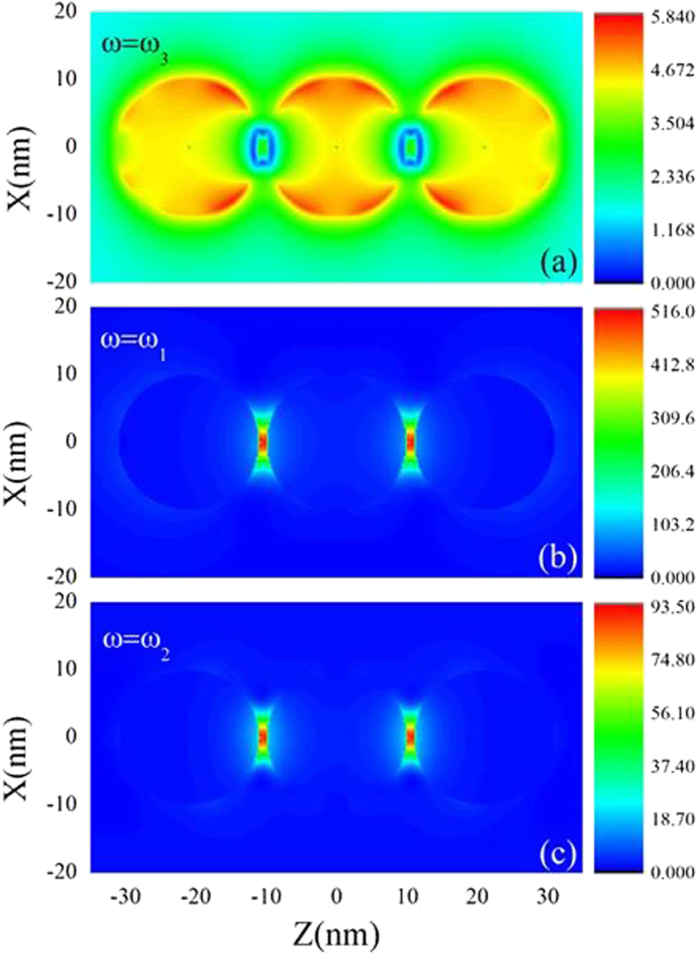
Electric field amplitude pattern of a linearly arranged silver nanosphere trimer with d = 1 nm at *ω*_3_ = 5470 THz (a), *ω*_1_ = 4530 THz (b) and *ω*_2_ = 4800 THz (c) for the normal incidence, which correspond to three peaks in Fig. 2**(a)**.

In order to further reveal two kinds of resonant properties, in Fig. 3 we show the comparison of local electric filed intensity in XZ plane at three different wavelengths (corresponding to three peaks) for the case with d = 1 nm. Figure 3(a) corresponds to the result of the single scattering resonance (*ω*_3_ = 5470 THz), localized surface plasmon excitation is around the single sphere and the fields in the gaps are not strong. In such a case, the interference term 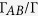 (red dotted line in Fig. 2(b)) between QEs is almost zero although the single QE spontaneous decay rate is very big. This is because the emissions of emitters are quenched when the emitter-sphere distances are very small. The cooperative behavior is destroyed by the nonradiative transition, and the large decay rate of the QE is due to dissipation of the metallic sphere, and the emission part is very small. Such a phenomenon has been pointed out in ref. 20. Thus, the entanglement between two QEs in such a case cannot be produced. In contrast, the electric fields mainly focus in the gap regions for the coupling resonances as shown in Fig. 3(a,b) (corresponding to peaks (1) and (2) in Fig. 2(a)). The coupling resonance causes the big interference term between QEs, which directly leads to the strong interaction and the generation of large entanglement (>0.5) between QEs.

### Three-qubit entanglement

**Figure 4:**
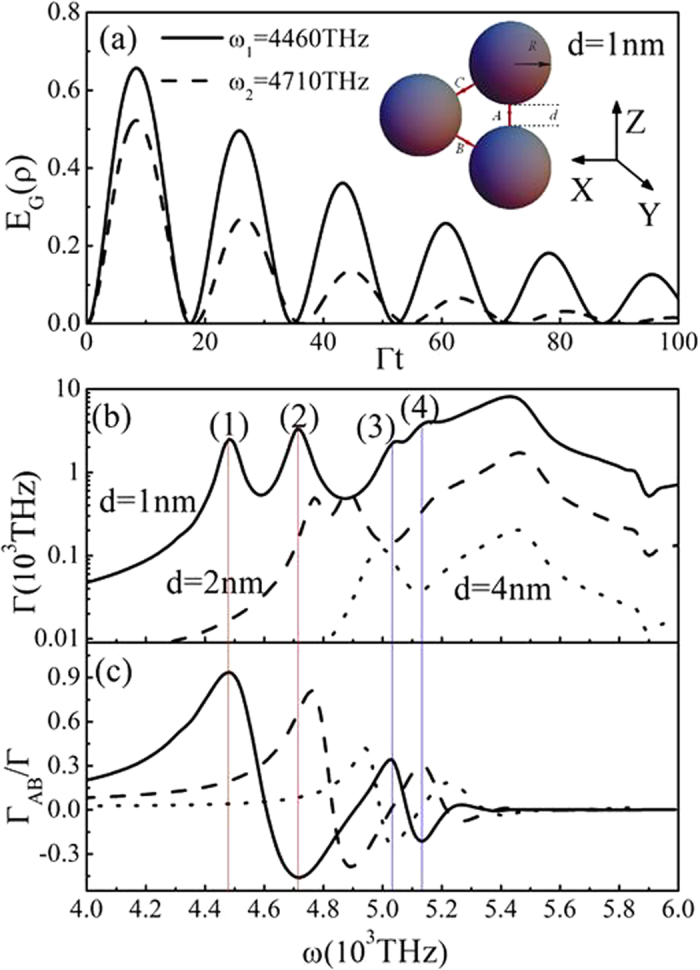
(**a**) Genuine entanglements of three QEs inserted in the hotspots of three spheres arranged in a triangle configuration showed in the inset at *ω*_1_ = 4460 THz and *ω*_2_ = 4710 THz. The corresponding spontaneous decay rate Γ (**b**) and interference term 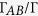 (**c**) of QEs with d = 1 nm (solid), d = 2 nm (dashed) and d = 4 nm (dotted), the other parameters are taken identical with those in Fig. 1.

If we consider three two-level QEs (A, B and C) located in the gaps of the metallic sphere cluster as shown in the inset of Fig. 4(a), three-qubit entanglement can be realized. Here, the orientations of the electric-dipole moments are all along the axis of any two spheres. The new states 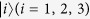 are taken as 
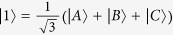
, 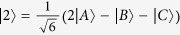 and 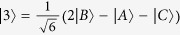. The decay rates of the three states are 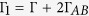 and 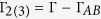. At *ω*_1_ = 4460 THz corresponding to the peak (1) marked in Fig. 4(b), 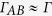, we obtain 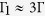 and 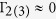, the density operator possesses the same form as Eq. (4) but 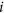 is replaced by 1, where the probability amplitude 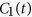 can be solved similar to the case for the two-QE regime. When 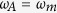, 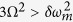, and with the initial condition 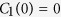, the solution is
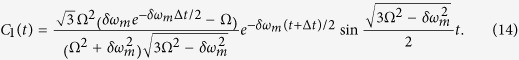


At *ω*_2_ = 4710 THz for the peak (2) in Fig. 4(b), 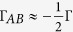, we have 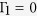 and 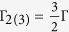, the density operator can also be expressed by Eq. (4) with *i* = 2(3). Similar to the procedure in solving 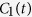, we can obtain
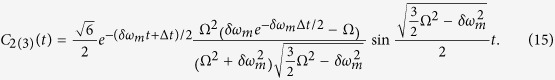


From Eqs (14 and 15) combining with Eqs (4, 5, 6), the genuine three-qubit entanglement can be obtained. The calculated results are shown in Fig. 4(a). The corresponding results for the single QE spontaneous decay rate Γ and the interference term 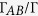 between QEs are given in Fig. 4(b,c), respectively. When d = 1 nm, 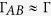 and 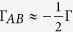 can be reached for the strong coupling condition. In such a case, large three-qubit entanglement (>0.5) can be observed clearly. However, with the increase of d, for example, when d = 2 nm or 4 nm, the single QE decay rate Γ becomes very small, and 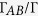 is also less than 0.8 for all frequencies, in these cases large three-qubit entanglements cannot be found. Compared with the two-qubit entanglement, the realization of three-qubit entanglement needs stronger resonance coupling, that is, the smaller gaps. With the decrease of d, we have found more peaks appear, such as peaks (3) and (4) marked in Fig. 4(b), which are caused by the collective resonance of three spheres. However considerable entanglement at the frequencies corresponding to these peaks cannot be produced because 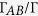 is very small as shown in Fig. 4(c). Therefore, to achieve large multi-qubit entanglement, two conditions, large Γ and 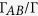, must be satisfied simultaneously.

### Four-qubit entanglement

**Figure 5:**
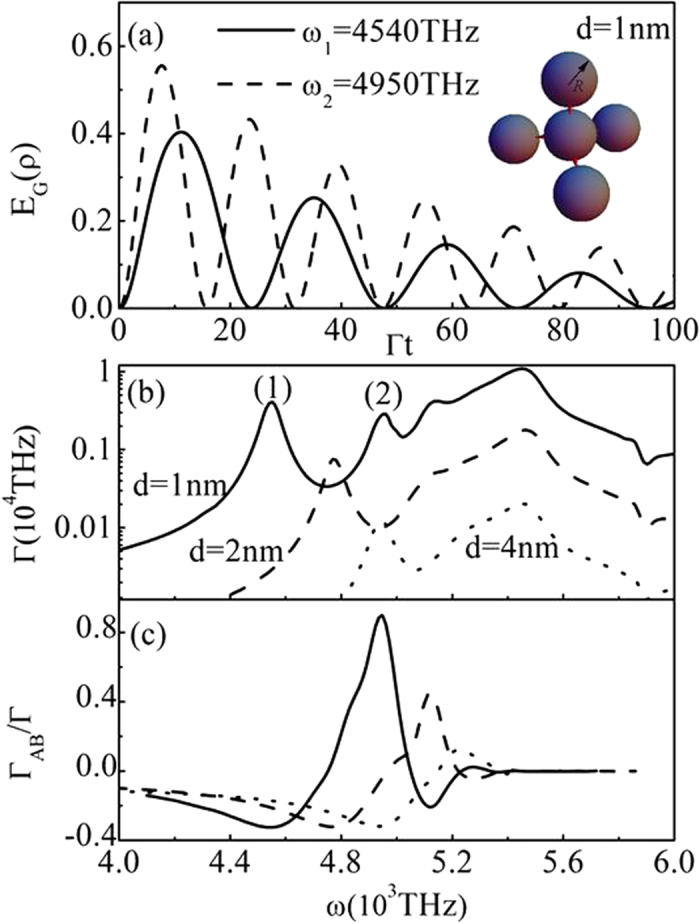
(**a**) Genuine entanglements of four QEs inserted in the hotspots of five spheres cluster as showed in the inset at *ω*_1_ = 4540 THz and *ω*_2_ = 4950 THz. The corresponding spontaneous decay rate Γ (**b**) and interference term 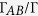 (**c**) of QEs with d = 1 nm (solid), d = 2 nm (dashed) and d = 4 nm (dotted), the other parameters are taken identical with those in Fig. 1.

Similar to the two-qubit and three-qubit cases, we can also realize four-qubit entanglement. We consider four two-level QEs (A, B, C and D) located in the gaps of the metallic sphere cluster as shown in the inset of Fig. 5(a), to ensure the symmetry, the four spheres are in the vertex of a tetrahedron, with another sphere in the center. The orientations of the electric-dipole moments are all along the axis of any two spheres. The new states 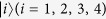 are taken as 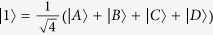, 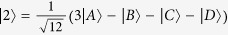, 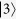 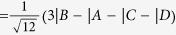 and 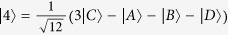. The decay rates of the four states satisfy 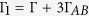 and 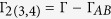. At *ω*_1_ = 4540 THz corresponding to the peak (1) in Fig. 5(b) for d = 1 nm, 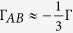, we obtain 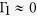 and 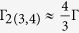, the density operator is expressed by Eq. (4) with *i* = 2(3, 4), in which 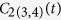 are given as
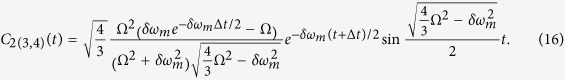
When the frequency is taken corresponding to the peak (2) in Fig. 5(b), 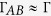, we have 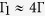 and 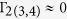, the density operator can be written as Eq. (4) with *i* = 1, and 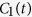 can be solved as
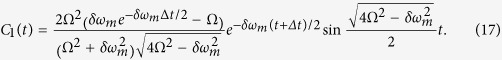
Figure 5(a) shows the calculated results for the genuine four-qubit entanglement at d = 1 nm from Eqs (16,17) and (4, 5, 6). The corresponding results for the single QE spontaneous decay rate Γ and the interference term 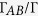 between QEs are given in Fig. 5(b,c), respectively. Similar to the case of three QEs, when the gaps become very small such as d = 1 nm, the conditions: 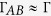 and 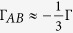 for large four-qubit entanglement are satisfied. With the increase of d, both Γ and 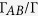 decrease rapidly, the corresponding entanglement is very small. In fact, the entanglement properties for the above systems depend on many factors such as the dimensions of metallic spheres, the separation distances between two spheres, the material properties of spheres and so on. Among these factors, the separation distances (gaps) are the most important as have been shown in the above discussions.

The above discussions only focus on two-, three- and four-qubit cases. In fact, our theory is suitable for designing any multi-qubit entanglement based on plasmonic hotspots. In the previous studies on the two-qubit entanglement mediated by one-dimensional plasmonic waveguides^35^,^37^, it has been pointed out that a continuous laser pumping can be used to have a stationary state with a high degree of entanglement. In the present cases, similar method can also be used.

Such a multi-qubit entanglement exhibits many advantages in comparing with other schemes for achieving entanglement. For example, it not only possesses longer duration of quantum beats, it is easy to be realized. Recently, some clusters of gold nanospheres, i.e. the dimers/trimers/tetramers, were fabricated successfully by using cysteine chiral molecules as linkers at the hotspots^19^. We expect our design for the multi-qubit entanglement can be realized and the phenomenon can be observed experimentally in the future.

## Conclusions

We have presented a theoretical method to study the strong coupling between an ensemble of QEs and surface plasmons excited by the nanoparticle cluster using the rigorous first-principles electromagnetic Green’s tensor technique. The method is suitable for designing any multi-qubit entanglement for two level QEs, although our discussions focus on two-, three- and four-qubit cases. Such a method for achieving multi-qubit entanglements exhibits many advantages in comparing with other schemes. For example, the multi-qubit entanglement for two-level QEs can be produced at different frequencies simultaneously, when they locate in the hotspots of metallic nanoparticle clusters. The duration of quantum beats for such an entanglement can reach two orders longer than that for the entanglement in the photonic cavity. The phenomena originate from collective excitations of coupling resonances in the cluster. In contrast to some previous investigations, we have also found that the entanglement between two QEs cannot be produced at the resonance excitation of the single scattering although the single QE spontaneous decay rate is very big. Potential applications of the present phenomena to the quantum-information processing are anticipated.

## Methods

### Dyadic Green’s function in the nanoparticle cluster by T-matrix method

The classical Green tensor in Eq. (2) can be calculated by the T-matrix method. The dyadic Green’s function 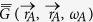 represents the electric field in the nanoparticle cluster excited by a unit dipole. In the framework of the T-matrix approach^53^,^54^,^55^, the incident and scattered fields are expanded in vector spherical functions (VSFs):
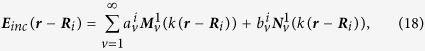

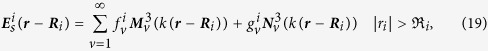
where 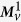, 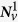, 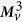 and 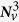 are the well-known VSFs, and 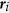 is a position vector in the coordinate of the *ith* particle. 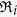 is radius of the smallest sphere circumscribing the *ith* object. 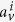, 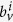, 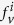 and 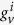 are the expansion coefficients, which can be readily known as soon as the form of the incident wave is given the *v* stands for (m, n) which are the indices of spherical harmonic functions. At the same time the internal field of the *ith* nanoparticles are written as,
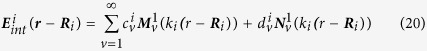


According to the T-matrix method^53^,^54^,^55^, 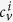 and 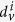 are related with 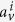 and 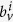 by the following matrix equation:
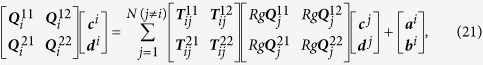
where 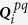 and 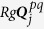 are the T-matrix blocks for the *ith* and *jth* particles, and 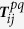 is block of the transition matrix between the *ith* particle and the *jth* particle^53^,^54^,^55^. By solving these equations, expansion coefficients of the inner field for each object can be known. And also according to the equation:
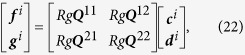
the scattering expansion coefficients 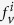 and 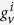 of each particle can be easily calculated, and the detailed forms of matrix elements 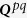 have been given in the supplementary materials. The field outside the circumscribing spheres then can be obtained using the following equation:
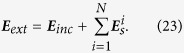


About the calculation of 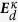 induced by a dipole with a momentum of 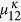, we take the exciting source as a dipole 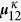 located in 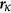, the incident wave can be expressed as
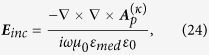
where
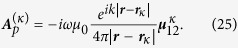
Expanding Eq. (24) to the same form of Eq. (18), from Eqs (19, 20, 21, 22, 23) we can obtain the external scattering field 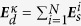 caused by the dipole. Then, the dyadic Green’s function 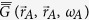 can be obtained.

### Solving eigenvalue of the system

For an arbitrary scatterer, the eigen-frequency is a complex 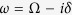, Ω and *δ* represent the center frequency and the line-width, respectively. They can be obtained from the following procedure. The relation between the scattering coefficients (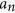 and 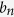) and the incident coefficients (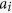 and 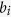) can be expressed as
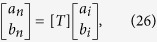
where [*T*] represents the scattering matrix. Multiplying the inverse matrix of [*T*] on both sides of Eq. (26), and let the incident coefficients be equal to zero, we arrive at
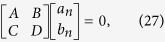
where
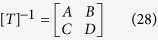
is the coefficient matrix of the system. The determinant of the matrix in Eq. (28) must be equal to zero if the Eq. (27) has non-trivial solutions. By using this condition, we can obtain the real and imaginary parts of the eigen-frequency. Therefore, the key problem is to construct the coefficient matrix. Based on the method of T matrix, the coefficient matrix for the nanoparticle cluster can be expressed as^53^
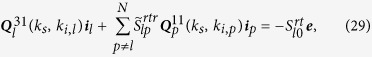
where 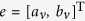 are the coefficients of incident field in the global coordinate, 
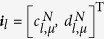
are the coefficients of internal field of the *lth* sphere in the local coordinate. The Q matrix is
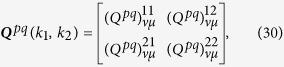
where 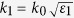 and 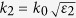. The expressions for the matrix elements: 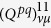 , 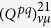, 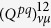 and 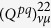 are given in Supplementary materials.

## Additional Information

**How to cite this article**: Ren, J. *et al.* Multifrequency multi-qubit entanglement based on plasmonic hot spots. *Sci. Rep.* **5**, 13941; doi: 10.1038/srep13941 (2015).

## Supplementary Material

Supplementary Information
