# Supplementary material for: Multifrequency multi-qubit entanglement based on plasmonic hot spots
Source: Sci Rep. 2015 Sep 9;5:13941. doi: 10.1038/srep13941 (PMC4563568; doi:10.1038/srep13941)
Supplement: Supplementary Information [file srep13941-s1.pdf]

Supplementary materials

Multifrequency multi-qubit entanglement based on plasmonic hot spots

Jun Ren, Tong Wu, and Xiangdong Zhang

According to the Ref. S1, the matrix elements $(Q^{pq})_{\nu\mu}^{11}$, $(Q^{pq})_{\nu\mu}^{21}$, $(Q^{pq})_{\nu\mu}^{12}$ and $(Q^{pq})_{\nu\mu}^{22}$ are expressed in the following:

$$(Q^{pq})_{\nu\mu}^{11} = \frac{\mathrm{j}k_1^2}{\pi}\int_S \{[\boldsymbol{n}(\boldsymbol{r}')\times \boldsymbol{M}_\mu^q(k_2\boldsymbol{r}')]\cdot \boldsymbol{N}_{\bar{\nu}}^p(k_1\boldsymbol{r}') + \sqrt{\frac{\varepsilon_2}{\varepsilon_1}}[\boldsymbol{n}(\boldsymbol{r}')\times \boldsymbol{N}_\mu^q(k_2\boldsymbol{r}')]\cdot \boldsymbol{M}_{\bar{\nu}}^p(k_1\boldsymbol{r}')\}\,\mathrm{d}S(\boldsymbol{r}'), \tag{S1}$$

$$(Q^{pq})_{\nu\mu}^{12} = \frac{\mathrm{j}k_1^2}{\pi}\int_S \{[\boldsymbol{n}(\boldsymbol{r}')\times \boldsymbol{N}_\mu^q(k_2\boldsymbol{r}')]\cdot \boldsymbol{N}_{\bar{\nu}}^p(k_1\boldsymbol{r}') + \sqrt{\frac{\varepsilon_2}{\varepsilon_1}}[\boldsymbol{n}(\boldsymbol{r}')\times \boldsymbol{M}_\mu^q(k_2\boldsymbol{r}')]\cdot \boldsymbol{M}_{\bar{\nu}}^p(k_1\boldsymbol{r}')\}\,\mathrm{d}S(\boldsymbol{r}'), \tag{S2}$$

$$(Q^{pq})_{\nu\mu}^{21} = \frac{\mathrm{j}k_1^2}{\pi}\int_S \{[\boldsymbol{n}(\boldsymbol{r}')\times \boldsymbol{M}_\mu^q(k_2\boldsymbol{r}')]\cdot \boldsymbol{M}_{\bar{\nu}}^p(k_1\boldsymbol{r}') + \sqrt{\frac{\varepsilon_2}{\varepsilon_1}}[\boldsymbol{n}(\boldsymbol{r}')\times \boldsymbol{N}_\mu^q(k_2\boldsymbol{r}')]\cdot \boldsymbol{N}_{\bar{\nu}}^p(k_1\boldsymbol{r}')\}\,\mathrm{d}S(\boldsymbol{r}'), \tag{S3}$$

$$(Q^{pq})_{\nu\mu}^{22} = \frac{\mathrm{j}k_1^2}{\pi}\int_S \{[\boldsymbol{n}(\boldsymbol{r}')\times \boldsymbol{N}_\mu^q(k_2\boldsymbol{r}')]\cdot \boldsymbol{M}_{\bar{\nu}}^p(k_1\boldsymbol{r}') + \sqrt{\frac{\varepsilon_2}{\varepsilon_1}}[\boldsymbol{n}(\boldsymbol{r}')\times \boldsymbol{M}_\mu^q(k_2\boldsymbol{r}')]\cdot \boldsymbol{N}_{\bar{\nu}}^p(k_1\boldsymbol{r}')\}\,\mathrm{d}S(\boldsymbol{r}'). \tag{S4}$$

The line-width δ and Rabi-frequency Ω for the two-, three- and four-qubit cases of the systems at $d = 1nm$ are given in the following tables:

Table 1 Two-qubit cases in Fig. 2 at $d = 1nm$

Peak in Fig. 2(a)	ω (THz)	Γ (THz)	δ (THz)	Ω (THz)
1	4530	2540	111	751
2	4800	3290	68.4	671
3	5470	13500	57.7	1250

Table 2 Three-qubit cases in Fig. 4 at $d = 1nm$

Peak in Fig. 4(b)	ω (THz)	Γ (THz)	δ (THz)	Ω (THz)
1	4460	2240	48.6	467
2	4710	3300	134	940
3	5440	8080	59.3	979

Table 3 Four-qubit cases in Fig. 5 at $d = 1nm$

Peak in Fig. 5(b)	ω (THz)	Γ (THz)	δ (THz)	Ω (THz)
1	4540	3820	100	874
2	4950	2850	56.2	566
3	5440	10800	59.3	1130

References

[S1]. Doicu, A., Wriedt, T. & Eremin, Y. A. *Light Scattering by Systems of Particles*. (Springer-Verlag, Berlin, Heidelberg, 2006).
